# Validation of the Indonesian version of the foot and ankle score in patients with chronic lateral ankle instability

**DOI:** 10.1186/s13047-021-00488-2

**Published:** 2021-08-04

**Authors:** I Putu Gde Surya Adhitya, Wen-Yu Yu, Putu Ayu Sita Saraswati, I Made Niko Winaya, Mau-Roung Lin

**Affiliations:** 1 Graduate Institute of Injury Prevention and Control, College of Public Health, Taipei Medical University, 250 Wu-Hsing Street, 11031 Taipei, Taiwan, Republic of China; 2 Department of Physical Therapy, College of Medicine, Universitas Udayana, P.B Sudirman Street, Bali 80232 Denpasar, Indonesia; 3 Department of Emergency Medicine, Taipei Medical University Hospital, 252 Wu-Hsing Street, 11031 Taipei, Taiwan, Republic of China

**Keywords:** Chronic lateral ankle instability, FAOS, Indonesia, Quality of life, Psychometrics

## Abstract

**Background:**

This study aimed to examine the psychometric performance of the Foot and Ankle Outcome Score (FAOS) used in Indonesian patients with chronic lateral ankle instability (CLAI).

**Methods:**

The FAOS was translated into Indonesian through standardized procedures. Among 224 patients with unilateral CLAI recruited from 14 physical therapy clinics during a 1-year period, reliabilities, construct validities, and responsiveness levels of the FAOS were examined. Active and passive range of motion of ankle dorsiflexion or plantiflexion, figure-of-eight, numeric pain rating scale (NPRS), and Short Form (SF)-36 were used to test the construct validities.

**Results:**

The five subscales indicated adequate internal consistency (Cronbach’s alpha, 0.74 ~ 0.96) and interrater test-retest reliabilities (interclass correlation coefficients, 0.80 ~ 0.94). Subscales of the FAOS moderately converged with those selected measures with similar constructs ($r$ values, 0.32 ~ 0.53), with the exception of the correlation of pain with the NPRS ($r$, -0.06). Results of the principal component analysis showed that the five-factor structure of the FAOS was appropriate for the Indonesian data, although six items (four in the pain and two in the other symptoms (OSs) subscales) did not perfectly fit their original subscales. Guyatt’s responsiveness index for the FAOS’s subscales changed in the SF-36’s physical function over a 1-month period and ranged 0.37 to 1.27.

**Conclusions:**

The Indonesian version of the FAOS demonstrated acceptable reliabilities and responsiveness, and fair construct validities among CLAI patients, although certain items in the pain and OSs subscales may need to be further explored and improved.

## Background

Lateral ankle sprains are the most common musculoskeletal injury, and chronic lateral ankle instability (CLAI) represents a repetitive lateral ankle sprain with various persistent symptoms, such as intermittent pain, swelling, ankle instability, and a restricted range of motion (ROM) [1, 2]. Insufficient treatment and outcome evaluations of initial ankle injuries often cause CLAI.

To evaluate the effectiveness and safety of a medical intervention for patients, patient-reported outcome measures, such as health-related quality of life (HRQOL), are considered to capture the patient’s perspective, thereby adding another dimension to understanding a patient’s response to treatment that cannot be extrapolated from physiologic endpoints [3]. HRQOL measures are used to evaluate a patient’s perspective on his/her activities of daily living, disabilities, and impairment [4, 5]. One of the most common HRQOL measures for the foot and ankle joint problems is the Foot and Ankle Outcome Score (FAOS), originally adapted from the Knee Injury and Osteoarthritis Outcome Score (KOOS) [6]. The FAOS is used to evaluate and monitor clinical outcomes after rehabilitation programs and other interventions for CLAI patients [7, 8].

The FAOS was initially developed in English and has been validated in several languages among patients with various foot- and ankle-related injuries [9–11]. However, the FAOS has not been validated in any Indonesian patients. Considering that the incidence and prevalence of ankle sprain injuries have dramatically increased in Indonesia, the fourth largest country in the world by population [12] and that an Indonesian version of the FAOS should be beneficial in maximizing clinical use by Indonesian patients, its cross-cultural validation is necessary. Furthermore, responsiveness is the ability of a scale to detect small but important changes over time [13]. In the absence of perfect validity, responsiveness should be considered a psychometric characteristic separate from the reliability and validity [5]. Nonetheless, few studies have investigated the responsiveness of the FAOS.

To validate and expand the use of the FAOS, this study examined its psychometric properties of score distributions, reliabilities, construct validities, and responsiveness in Indonesian patients with CLAI.

## Methods

### Participants

During the year 2019, we recruited study participants from 14 physical therapy (PT) clinics, situated in 14 major provinces (Bali, Banten, Jakarta, Jawa Barat, Jawa Tengah, Jawa Timur, Kalimantan Selatan, Nusa Tenggara Barat, Papua Barat, Riau, Sulawesi Selatan, Sumatra Barat, Sumatra Selatan, and Yogyakarta) in Indonesia. Eligible participants were aged ≥ 17 years who had been diagnosed with unilateral CLAI. The criteria for identifying unilateral CLAI consisted of one-sided, repetitive lateral ankle sprains that had occurred at least 6 weeks before the assessment, a positive anterior drawer test (an anterior translation more significant than 1 cm on the injured side vs. the uninjured side), a positive talar tilt test (an inversion tilt of the talus of ≥ 9° on the injured side vs. the uninjured side), and having experienced residual symptoms such as a subjective feeling of giving way [1, 2]. We excluded individuals who had bilateral CLAI, a bone fracture, muscle strain, other ligament sprains on the lower limbs, or who could not understand the study questions. In total, 224 patients with a unilateral CLAI agreed to participate in the study, and written informed consent was obtained from each participant.

The study was approved by the Ethical Committee of the Institutional Review Board College of Medicine, Universitas Udayana/Central Public Hospital Sanglah Denpasar (permission no. 60/UN14.2.2.VII.14/LP/2019).

### FAOS

The original version of the FAOS consists of 42 items across five subscales, including pain (nine items, P1 ~ P9), other symptoms (OSs) (seven items, OS1 ~ OS7), activities of daily living (ADLs) (17 items, ADL1 ~ ADL17), sports and recreational function (SRF) (five items, SRF1 ~ SRF5), and foot- and ankle-related quality of life (FAQL) (four items, FAQL1 ~ FAQL4) [6]. Each item assesses an individual’s ability in the last week and is rated on a 5-point Likert scale ranging from 0 to 4 (0, none; 1, mild; 2, moderate; 3, severe; and 4, extreme problems). Missing values were replaced by the mean value of the subscale when two or fewer items were missing on a subscale; and there was no score for the subscale when more than two items were missing. Each subscale score was calculated independently using the mean score of each subscale divided by a factor of 4. The possible score ranges from 0 to 100, with a higher score representing fewer problems in the foot and ankle.

### Translation

According to standard international guidelines of cross-cultural adaptation [14], the English version of the FAOS was translated into an Indonesian version through four steps. First, two forward translations from English to Indonesian were independently produced by two bilingual translators with different backgrounds (PT and computer science), who speak Indonesian as their mother tongue. The two translators synthesized their translations and achieved consensus via face-to-face discussions. Then, backward translations from Indonesian to English were performed independently by two bilingual translators whose mother tongue was English. Then, two focus groups sessions were conducted with two physical therapists, two translators, an orthopedist, a physiatrist, and a language professional to harmonize the meaning of the pre-final Indonesian version of the FAOS. Finally, a pilot test of the pre-final version was conducted among 40 CLAI patients to check whether the questionnaire could be comprehended in real field environments.

### Validation procedures

Each participant received an initial assessment, including active ROM (AROM), passive ROM (PROM), and figure-of-eight, while also completing a set of questionnaires, including the FAOS, a numeric pain rating scale (NPRS), and Short Form-36 (SF-36). To evaluate the test-retest reliability, 56 participants (four randomly selected from each province) were re-administered the FAOS 3 days after the initial assessment. To evaluate the responsiveness, 60 patients in Bali Province who received PT treatment for CLAI two times per week for 1 month were also re-administered the FAOS after completing the intervention. All assessors, procedures of data collection, the interview process and interviewers’ attitudes, instrument administration, and physical assessments were standardized and equalized through participation in a 4-h online training course.

### Physical assessments and instruments

#### AROM and PROM

The AROM and PROM measure how far the joint can be moved by the patient and the physical therapist, respectively [15]. Measurements of dorsiflexion and plantiflexion were obtained with a 30-cm 360°-goniometer marked in 1° increments while patients actively and passively moved their ankle joints [16]. Three trials for each of the AROM and PROM measurements on both ankles were obtained, with the average as the representative value. When a difference between the uninjured and injured ankles for each of the AROM and PROM measurements was > 3°, the ankle was considered to be restricted [17].

#### Figure-of-eight

The figure-of-eight assesses ankle swelling using retractable plastic tape that is marked in 1-cm increments. The tape was bandaged around the ankle in a figure-of-eight pattern applied at the insertion of the tibialis anterior tendon, the base of the 5th metatarsal, the navicular tuberosity, and the medial and lateral malleoli as anatomical landmarks [18]. If the difference between the uninjured and injured ankles exceeded 1.26 cm, a swollen ankle was recorded [19]. Three trials for the measurement on both ankles were conducted, and the average of each ankle was used as the representative value.

#### NPRS

The NPRS measures pain severity, in which an individual rates his/her latest sensation of pain on an 11-point numeric scale. Numbers from 0 to 10 on the scale represent “no pain” to “the worst pain”. Each participant was asked to rate his/her ankle pain from three aspects, including current pain, worst pain, and least pain, in the past 24 h; the average of the three was used to represent ankle pain [20].

#### SF-36

The SF-36 is the most commonly used generic HRQOL instrument [21, 22], which consists of 36 items across eight subscales of physical function (PF), role physical (RP), bodily pain (BP), general health (GH), mental health (MH), role emotion (RE), social function (SF), and vitality (VT). Each subscale score ranged from 0 to 100, with a higher score indicating better life quality.

### Statistical analysis

#### Score distributions

A high ceiling or floor effect on each subscale of the FAOS can make the FAOS difficult to distinguish patients from one another. The presence of a high ceiling or floor effect was considered when > 15 % of participants endorsed the highest or lowest possible score [23].

#### Reliabilities

The internal consistency of each subscale of the FAOS was determined using Cronbach’s alpha, which represents the extent to which the items of the subscale measure a similar construct. Cronbach’s alpha coefficients of ≥ 0.7 were considered acceptable. The interrater test-retest reliability for each of the subscales was determined using the interclass correlation coefficient (ICC) by evaluating whether the subscale was able to assess participants’ conditions consistently over a 3-day interval. ICCs of > 0.7 were considered satisfactory [10].

#### Construct validities

Convergent validity was determined by computing Spearman’s correlation coefficients of the five FAOS subscales with the AROM, PROM, figure-of-eight, NPRS, and eight SF-36 subscales. Correlation coefficients of < 0.3, 0.3 ~ 0.6, and > 0.6 were respectively considered to indicate low, moderate, and high relationships [11]. Based on previous findings [1, 11], we hypothesized moderate/high correlations of the FAOS’s pain subscale with the NPRS and SF-36’s BP; of the FAOS’s OSs with the PROM and figure-of-eight; of the FAOS’s ADLs with the AROM and SF-36’s PF; of the FAOS’s SRF with the figure-of-eight and SF-36’s PF; and of the FAOS’s FAQL with the AROM and SF-36’s BP. Construct validity of the FAOS was considered as acceptable when > 75 % of all hypotheses or correlations formulated as described above were confirmed [24].

The Kaiser-Meyer-Olkin Measure of Sampling Adequacy and Bartlett’s Test of Sphericity were used to assess the suitability of the data for a factor analysis. A principal component analysis (PCA) as the extraction method and the Promax with Kaiser Normalization as the rotation method were conducted to examine the five-component structure of the FAOS. The number of factors was forced to be five in order to validate whether the Indonesian version of the FAOS showed the same five-component structure as the original FAOS. Those items with a factor loading of ≥ 0.35 were considered satisfactory [25].

#### Responsiveness

One month after the baseline assessment, 60 patients in Bali Province who received PT were followed-up, and the FAOS and the “change” item of the SF-36 (an attempt to measure the change in the HRQOL over 1 year) were re-administered at that time. We applied both distribution-based and anchored-based approaches to evaluate the responsiveness of the FAOS. Cohen’s effect sizes were calculated by dividing the mean change of each FAOS subscale with the estimated standard deviation (SD) before and after the intervention [13]. The time frame of the SF-36’s “change” item was modified to be 1 month instead of 1 year (i.e., “Compared to 1 month ago, how would you rate your general health now?“*).* The response to this item was used as an external reference to estimate the anchored-based responsiveness. According to Guyatt’s method [5], the anchor-based responsiveness of the FAOS was calculated as the mean change in a subscale score over the 1-month period for participants who improved or deteriorated according to the SF-36’s “change” item, divided by the SD of the change in that subscale for those unchanged. Effect sizes of 0.2 ~ 0.5, 0.5 ~ 0.8, and > 0.8 in responsiveness were respectively considered to be small, moderate, and large [26]. An effect size of 0.2 indicated a clinically important difference [27]. All statistical analyses were performed using SPSS vers. 24 statistical software (IBM Corporation, Armonk, NY).

## Results

### Participant characteristics

As shown in Table 1, among the 224 participants, the mean age was 23.2 years and the time since injury was 15.8 months; 156 (69.6 %) were men and 68 (30.4 %) were women; 69 (30.8 %) had attained a college education or above; and 60 (26.8 %) were obese. Furthermore, 124 (55.4 %) of the participants had sustained a CLAI on the right leg, 152 (67.9 %) had ankle swelling, and the dorsiflexion AROM was restricted in 158 (70.5 %), the plantiflexion AROM was restricted in 152 (67.9 %), the dorsiflexion PROM was restricted in 137 (61.2 %), and the plantiflexion PROM was restricted in 127 (56.7 %). The mean score of the NPRS was 4.1 points, and those of the SF-36’s eight subscales varied from 54.5 to 74.5 points.

**Table 1:** Demographic and clinical characteristics of 224 patients with chronic lateral ankle instability

Characteristic	Mean$\pm$SD or *n* (%)	
Age (years)	23.2$\pm$7.7	
Time since injury (months)	15.8$\pm$14.1	
Se		
Male	156 (69.6)	
Female	68 (30.4)	
Educational level		
College or above	69 (30.8)	
High school	146 (65.2)	
Junior high school	6 (2.7)	
Elementary or below	3 (1.3)	
Body-mass index		
Normal	104 (46.4)	
Underweight	28 (12.5)	
Overweight	32 (14.3)	
Obese	60 (26.8)	
Injured leg, right	124 (55.4)	
Swelling present	152 (67.9)	
Active range of motion		
Dorsiflexion, restricted	158 (70.5)	
Plantiflexion, restricted	152 (67.9)	
Passive range of motion		
Dorsiflexion, restricted	137 (61.2)	
Plantiflexion, restricted	127 (56.7)	
Numeric pain rating scale	4.1$\pm$2.1	
Short Form-36		
Physical function	74.5$\pm$21.6	
Role physical	54.5$\pm$32.8	
Bodily pain	73.6$\pm$20.9	
General health	64.3$\pm$16.7	
Mental health	68.2$\pm$18.4	
Role emotional	56.6$\pm$35.5	
Social functioning	73.9$\pm$21.3	
Vitality	65.9$\pm$18.8	

*SD* standard deviation

### Translation

During the translation process, the expert committee changed the word *“*grinding*”* to *“*friction*”* in an item of pre-final FAOS (“Do you feel grinding, hear a clicking or any other type of noise when your foot/ankle moves?“), because *“*grinding*”* is not a common word used in the medical community but a term in the food industry in Indonesia. In the pilot test, those 40 patients with a CLAI expressed that they comprehended the 42 questions in the pre-final FAOS with no difficulty.

### Score distributions

**Fig. 1:**
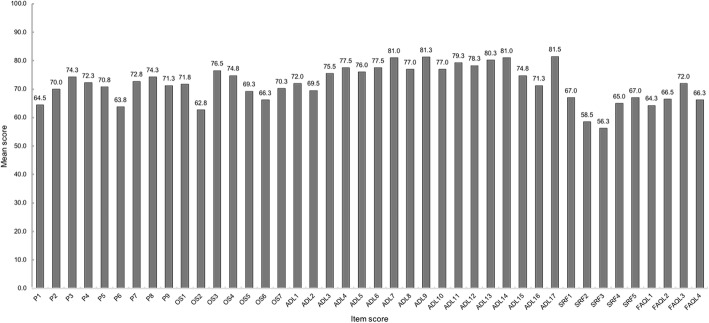
Mean item scores of the Foot and Ankle Outcome Score among 224 patients with chronic lateral ankle instability

As shown in Fig. 1, the means of item scores of the FAOS varied from the lowest at 56.3 points (SRF3) to the highest at 81.5 points (ADL17). As shown in Table 2, among the five subscales of the FAOS, the percentage of the floor and ceiling values ranged from 0.0 to 0.9 % and from 1.3 to 7.6 %, respectively. No high ceiling or floor effect for any of the subscales was found.

**Table 2:** Score distributions, Cronbach’s alpha, and intraclass correlation coefficients for the Indonesian version of the FAOS

FAOS subscale	No. of items	Mean$\pm$SD	Median (range)	% floor values	% ceiling values	Cronbach’s alpha	ICC
Pain	9	70.4$\pm$15.4	72 (25 ~ 100)	0.0	1.3	0.87	0.87
OSs	7	70.2$\pm$18.0	71 (21 ~ 100)	0.0	2.7	0.77	0.80
ADLs	17	77.1$\pm$17.8	78 (15 ~ 100)	0.0	7.6	0.96	0.90
SRF	5	62.8$\pm$23.2	65 (5 ~ 100)	0.0	3.6	0.91	0.94
FAQL	4	67.4$\pm$21.8	69 (0 ~ 100)	0.9	7.6	0.74	0.86

*ADLs* activities of daily living, *FAOS* foot and ankle outcome score, *FAQL* foot- and ankle-related quality of life, *ICC* interclass correlation coefficient, *OSs* other symptoms, *SD* standard deviation, *SRF* sports and recreational function

### Reliabilities

As to internal consistencies of the five subscales of the FAOS, Cronbach’s alpha coefficients ranged 0.74 ~ 0.96 (Table 2). For the interrater test-retest reliability, ICCs ranged 0.80 ~ 0.94, among which the FAQL displayed the highest ICC (0.95) and OSs had the lowest ICC (0.89).

### Construct validities

As shown in Table 3, consistent with our hypotheses, moderate/high correlations existed between the FAOS’s subscales and the ankle dorsiflexion/plantiflexion AROM, figure-of-eight, NPRS, and SF-36’s subscales, with *r* values ranging 0.32 ~ 0.53. Unexpectedly, there was a low correlation between the FAOS’s pain and the NPRS (r_s_ = -0.06).

**Table 3:** Spearman’s correlation coefficients between the Indonesian version of the FAOS and selected measures

FAOS subscale	Pain	OSs	ADLs	SRF	FAQL
Dorsiflexion AROM	-	-	0.53^†^	-	0.41^†^
Plantiflexion AROM	-	-	0.49^†^	-	0.40^†^
Dorsiflexion PROM	-	0.52^†^	-	-	-
Plantiflexion PROM	-	0.51^†^	-	-	-
Figure-of-eight	-	0.53^†^	-	0.32^†^	-
Numeric pain rating scale	-0.06	-	-	-	-
SF-36’s physical function	-	-	0.51^†^	0.42^†^	-
SF-36’s bodily pain	0.37^†^	-	-	-	0.40^†^

*ADLs* activities of daily living, *AROM* active range of motion, *FAOS* foot and ankle outcome score, *FAQL* foot- and ankle-related quality of life, *OSs* other symptoms, *PROM *passive range of motion, *SF-36* Short Form-36, *SRF* sports and recreational function

^†^*p* < 0.01

The Kaiser-Meyer-Olkin Measure of Sampling Adequacy was 0.942 and Bartlett’s Test of Sphericity was < 0.001, indicating that the PCA was suitable for our data (i.e., the proportion of variance was caused by underlying factors and the correlation matrix was not an identity matrix). Results of the PCA are shown in Table 4.

**Table 4:** Results of the principle component analysis to identify five factors of the 42-item FAOS

Item	Factor 1	Factor 2	Factor 3	Factor 4	Factor 5
P1	0.264	0.355	0.343	**0.115**	0.525
P2	0.166	0.382	0.351	**0.380**	0.307
P3	0.420	0.308	0.428	**0.256**	0.271
P4	0.350	0.368	0.423	**0.302**	0.215
P5	0.533	0.256	0.191	**0.311**	0.074
P6	0.385	0.282	0.258	**0.637**	0.064
P7	0.442	0.121	-0.011	**0.493**	0.132
P8	0.524	0.069	-0.070	**0.522**	0.059
P9	0.476	0.136	0.131	**0.626**	0.057
OS1	0.034	0.191	**0.556**	0.119	0.165
OS2	0.042	-0.070	**0.359**	0.354	0.381
OS3	0.073	0.078	**0.551**	0.242	0.198
OS4	0.274	0.090	**0.804**	-0.022	0.011
OS5	0.298	0.121	**0.797**	-0.078	0.012
OS6	0.469	0.234	**0.254**	0.316	0.147
OS7	0.523	0.261	**0.055**	0.247	0.162
ADL1	**0.522**	0.216	0.243	0.381	0.067
ADL2	**0.524**	0.304	0.325	0.356	0.083
ADL3	**0.741**	0.255	0.094	0.191	0.121
ADL4	**0.769**	0.199	0.149	0.292	0.080
ADL5	**0.751**	0.306	0.226	0.136	0.096
ADL6	**0.711**	0.250	0.271	0.238	-0.023
ADL7	**0.725**	0.287	0.077	0.171	0.021
ADL8	**0.688**	0.315	0.107	0.200	0.088
ADL9	**0.705**	0.226	0.213	0.066	0.155
ADL10	**0.724**	0.131	0.279	0.215	0.122
ADL11	**0.753**	0.261	0.221	-0.004	0.145
ADL12	**0.769**	0.108	0.187	0.145	0.224
ADL13	**0.853**	0.116	0.095	0.152	0.154
ADL14	**0.799**	-0.026	0.024	0.144	0.196
ADL15	**0.713**	0.211	0.097	0.079	0.309
ADL16	**0.554**	0.413	0.218	0.097	0.171
ADL17	**0.603**	0.226	0.205	0.163	0.136
SRF1	0.411	**0.638**	0.227	0.102	0.115
SRF2	0.201	**0.860**	0.111	0.132	0.069
SRF3	0.219	**0.861**	0.046	0.138	0.043
SRF4	0.312	**0.763**	0.145	0.099	0.210
SRF5	0.433	**0.622**	0.249	0.062	0.121
FAQL1	0.122	0.145	0.249	0.086	**0.643**
FAQL2	0.078	-0.002	-0.074	0.108	**0.776**
FAQL3	0.332	0.159	0.130	-0.076	**0.622**
FAQL4	0.420	0.353	0.337	0.127	**0.497**

*ADL* activities of daily living, *FAOS* foot and ankle outcome score, *FAQL *foot- and ankle-related quality of life, *OS* other symptoms, *P* pain, *SRF* sports and recreational function

^a^ Factor loadings in bold were expected to be ≥ 0.35

All items originally assigned to the three subscales of the ADLs, SRF, and FAQL displayed factor loadings of > 0.35. On the other hand, two items originally assigned to OSs (OS6 and OS7) and four items assigned to pain (P1, P3, P4 and P5) displayed loading factors of < 0.35.

### Responsiveness

Effect sizes on the responsiveness over the 1-month period for each subscale of the FAOS are shown in Table 5. The distribution-based Cohen’s effect sizes for the five FAOS subscales ranged 0.39 ~ 0.77, while score changes of the effect size of 0.2 for the pain, OSs, ADLs, SRF, and FAQL subscales were 4.46, 3.62, 1.95, 2.84, and 2.25, respectively. Of the 60 patients, 24 rated their condition “somewhat better” or “much better” than 1 month previous (improvement) and 15 rated their condition “somewhat worse” or “much worse” than 1 month previous (deterioration), with the remaining participants indicating that their condition was unchanged at 1 month after the intervention. The anchor-based effect sizes for the five FAOS subscales, based on a deterioration of the SF-36’s “change” item, ranged from − 0.31 to -1.27 and those based on an improvement of the item ranged from 0.63 to 1.13.

**Table 5:** Distribution-based and anchor-based responsiveness of the Indonesian FAOS subscales at 1 month

FAOS subscale	At the baseline mean ± SD	Changes at 1 month mean ± SD	Scores of effect size at 0.2	Cohen’s effect size	Changes in the unchanged group mean ± SD	Changes in the deteriorated group mean ± SD	Changes in the improved group mean ± SD	Guyatt’s responsiveness index	
								Deteriorated	Improved
Pain	64.7 ± 4.2	12.7 ± 14.8	4.46	0.77	9.4 ± 11.9	−8.2 ± 15.9	13.4 ± 17.6	−0.69	1.13
OSs	69.5 ± 17.7	12.8 ± 20.0	3.62	0.70	10.5 ± 17.0	−13.8±22.1	14.4 ± 24.5	−0.81	0.85
ADLs	75.5 ± 16.9	6.6 ± 14.5	1.95	0.40	3.7 ± 13.3	−4.9 ± 17.4	10.4 ± 19.6	−0.37	0.78
SRF	58.8 ± 23.8	13.5 ± 23.5	2.84	0.58	8.6 ± 19.7	−12.6 ± 22.9	20.2 ± 28.9	−0.64	1.03
FAQL	65.4 ± 19.9	9.0 ± 26.6	2.25	0.39	10.4 ± 21.5	−27.4 ± 27.9	13.6 ± 31.4	−1.27	0.63

*ADLs* activities of daily living, *FAOS* foot and ankle outcome score, *FAQL* foot- and ankle-related quality of life, *OSs* other symptoms, *SD* standard deviation, *SRF* sports and recreational function

## Discussion

An appropriate measure such as the FAOS is essential for monitoring patient outcomes and the efficacy of treatment in CLAI patients. Results of this study demonstrated that all five subscales of the Indonesian version of the FAOS displayed nearly symmetrical score distributions, low floor and ceiling values, acceptable internal consistency and test-retest reliability, satisfactory responsiveness, and fair construct validity, although certain items in the pain and OSs subscales showed lower factor loadings.

Among the five subscales of the FAOS, the ADLs and FAQL had the highest proportions of ceiling values, indicating that a substantial proportion of participants might not have experienced any restriction in their daily activities, and they may also have a satisfactory social life. Previous studies showed that young adults with CLAI maintained sufficient performance of their daily activities and an adequate quality of life [28], whereas older CLAI patients had poorer daily activities and quality of life compared to healthy older people [29]. Our study sample may have contributed to the high ceiling values in the FAOS’s ADL and FAQL in that young patients have faster physical and mental recovery from neuromuscular dysfunction, as well as a faster return to routine ADLs and sports, compared to older patients [30].

A low correlation unexpectedly existed between the pain subscale of the FAOS and the NPRS. A study even found a negative correlation (*r*=-0.74) between FAOS’s pain and the NPRS [11]. One possible explanation for this result is that the FAOS and NPRS use different time periods to assess pain. The FAOS measures pain based on multiple activities during the previous week, and the NPRS evaluates the intensity of pain in the past 24 h. Since pain is a multidimensional construct [31], the underlying constructs between the FAOS’s pain and NPRS might considerably differ. Future research might consider using other pain instruments, instead of the NPRS, with a construct similar to the FAOS’s pain subscale.

Item P1 (pain frequency) had a low loading on the latent pain subscale (Factor 4) but had a higher loading on the latent FAQL (Factor 5). This low loading might have been due to P1 evaluating the pain frequency, while the other items of the subscale evaluate the pain intensity for a specific ankle function. Another possible explanation for this finding is that participants may have misperceived P1, which asks about the extent to which the foot and ankle problem affects their daily life, like FAQL1, since other items of the pain subscale ask about the pain intensity in specific ADLs [32]. Items P3 (pain during ankle straightening), P4 (pain during ankle bending), and P5 (pain on walking on a flat surface) had low loadings onto their latent pain subscale but had higher loadings onto the latent ADLs or OSs factors from the PCA. Since young athletes usually have high expectations for returning to their preinjury sport and overcoming setbacks following an injury [33], the characteristics of our young participants may have played a role in that result. Furthermore, individuals with a higher athletic identity also had more-positive attitudes and reported a higher level of willingness to play through pain than did those with a lower athletic identity, and young athletes commonly have a strong athletic identity [34]. Since young Indonesian patients are more likely than older patients to undertake self-treatment with anti-inflammatory drugs to reduce musculoskeletal pain [35], those with a CLAI might use over-the-counter pain medications to keep physically and socially active in their daily lives or sport activities [36].

Items OS6 (morning stiffness) and OS7 (resting stiffness) were not moderately or highly correlated with OSs but were with ADLs. We noted that both OS6 and OS7 assess the severity of an ankle’s symptoms during a specific time, while the other items of the OSs assess the severity in a particular function. In addition, it seems that the type of rating scale anchors of OS6 and OS7 were the same as those of the FADL’s items (intensity type) but different from the other items of OSs (frequency type). Nonetheless, associations of morning stiffness with functional disability and pain were found to be stronger than the association of swelling and the erythrocyte sedimentation rate in patients with early rheumatoid arthritis given that morning stiffness is a symptom used as a measurement of rheumatoid arthritis [37]. As such, our participants might have perceived foot/ankle stiffness in the morning as a functional disability during daily living rather than as a biomarker of CLAI. Furthermore, since resting stiffness may result from a long period of immobilization and muscle positions [38], our participants may have perceived ankle resting stiffness during sedentary activities, such as studying, working, and watching movies.

Both the distribution-based and anchor-based responsiveness statistics indicated that the FAOS is a responsive HRQOL measure for CLAI patients. Among the five FAOS subscales, pain was the most responsive, possibly because pain is the most common problem of CLAI patients and often is the main reason for a clinical visit [1, 2]. Moreover, most effect sizes of the FAOS subscales in improvement of the SF-36’s “change” were larger than those in deterioration of the SF-36’s “change”, indicating that FAOS items were more sensitive to positive changes in the health status [39]. Anchor-based responsiveness depends on external measures, and here, it was determined by the strength of the association of the FAOS subscale with the SF-36’s “change”. Furthermore, a lower responsiveness in the distribution-based approach vs. the anchor-based approach could have resulted to some extent from positive changes occurring over a 1-month period, which were adjusted in anchor-based responsiveness, because those who perceived no change in the SF-36’s “change” item still displayed considerable positive changes in all five FAOS subscales.

There are some limitations to the study. First, the results possibly cannot be generalized to older populations with CLAI since the ages of our participants ranged 17 ~ 35 years, and they still maintained moderate to high levels of physical activity in their daily lives. It would be intriguing to further investigate whether the FAOS has age-related differential item functioning for CLAI patients. Second, examination of the construct validity was dependent on the selected measures, and some of those measures, such as the NPRS, might not be very appropriate, although the NPRS is one of the most commonly used tools to measure pain. It seems that the NPRS could not capture the complexity and idiosyncratic nature of the pain experiences of CLAI patients. Third, we did not measure intra-articular lesions of the ankle or physical functions of the hip and knee. Intra-articular lesions (synovitis, cartilage defects, tendinitis, and ligament tears) were suggested to be the cause of persistent symptoms of CLAI and were associated with clinical outcomes [40], while hip and knee problems might also affect ankle functions [41].

## Conclusions

The Indonesian version of the FAOS has reasonable score distributions, acceptable reliabilities and responsiveness, and fair construct validity among young CLAI patients, although certain items in the pain and OSs subscales may need to be further explored and improved.

## Data Availability

The data analyzed in this study are not publicly available. However, data are available from the authors upon reasonable request.

## Abbreviations

ADLs: Activities of daily living
AROM: Active ROM
BP: Bodily pain
CLAI: Chronic lateral ankle instability
FAOS: Foot and ankle outcome score
FAQL: Foot- and ankle-related quality of life
GH: General health
HRQOL: Health-related quality of life
ICC: Interclass correlation coefficient
KOOS: Knee injury and osteoarthritis outcome score
MH: Mental health
NPRS: Numeric pain rating scale
OSs: Other symptoms
PCA: Principal component analysis
PF: :Physical function
PROM: Passive ROM
RE: Role emotion
ROM: Range of motion
RP: Role physical
SD: Standard deviation
SF-36: Short Form-36
SF: Social function
SRF: Sports and recreational function
VT: Vitality
